# Biomarkers in Colorectal Cancer: Actual and Future Perspectives

**DOI:** 10.3390/ijms252111535

**Published:** 2024-10-27

**Authors:** Horia-Dan Lișcu, Nicolae Verga, Dimitrie-Ionuț Atasiei, Dumitru-Cristinel Badiu, Adrian Vasile Dumitru, Flavia Ultimescu, Christopher Pavel, Roxana-Elena Stefan, Diandra-Carmen Manole, Andreea-Iuliana Ionescu

**Affiliations:** 1 Discipline of Oncological Radiotherapy and Medical Imaging, Carol Davila University of Medicine and Pharmacy, 050474 Bucharest, Romania; horia-dan.liscu@drd.umfcd.ro (H.-D.L.); andreea-iuliana.miron@drd.umfcd.ro (A.-I.I.); 2 Radiotherapy Department, Colțea Clinical Hospital, 030167 Bucharest, Romania; nicolae.verga@umfcd.ro; 3 Department of Surgery, Bagdasar Arseni Clinical Emergency Hospital, Carol Davila University of Medicine and Pharmacy, 8 Eroii Sanitari Bd., 050474 Bucharest, Romania; dumitru.badiu@umfcd.ro; 4 Department of Pathology, University Emergency Hospital Bucharest, Carol Davila University of Medicine and Pharmacy, 8 Eroii Sanitari Bd., 050474 Bucharest, Romania; dr.adriandumitru@yahoo.com; 5 Department of Pathology, Institute of Oncology Alexandru Trestioreanu, Carol Davila University of Medicine and Pharmacy, 050474 Bucharest, Romania; flavia.ultimescu@drd.umfcd.ro; 6 Department of Gastroenterology, Floreasca Emergency Hospital Bucharest, Carol Davila University of Medicine and Pharmacy, 050474 Bucharest, Romania; christopher-jesse-vlad.pavel@drd.umfcd.ro; 7 General Surgery Department, Clinic of General and Esophageal Surgery, Sf. Maria Clinical Hospital, Carol Davila University of Medicine and Pharmacy, 050474 Bucharest, Romania; roxana-elena.stefan@drd.umfcd.ro; 8 Department of Endocrinology, Faculty of General Medicine, Carol Davila University of Medicine and Pharmacy, 8 Eroii Sanitari Bd., 050474 Bucharest, Romania; m.diandracarmen@gmail.com

**Keywords:** biomarkers, colorectal cancer, stool DNA, circulating tumor DNA, circulating tumor cell, gut microbiome

## Abstract

Biomarkers in colorectal cancer (CRC) are of great interest in the current literature due to improvements in techniques such as liquid biopsy and next-generation sequencing (NGS). However, screening methods vary globally, with multi-target stool DNA (mt-sDNA) predominantly used in the USA and, more recently, the Cologuard Plus; biomarkers such as the Galectins family and septins show promise in early detection. Gut microbiome assessments, such as Fusobacterium nucleatum, are under intense exploration. Diagnostic tests, such as circulating DNA analysis via NGS, exhibit effectiveness and are being increasingly adopted. Circulating tumor cells emerge as potential alternatives to traditional methods in terms of diagnosis and prognosis. Predictive biomarkers are well established in guidelines; nonetheless, with the aid of machine learning and artificial intelligence, these biomarkers may be improved. This review critically explores the actual dynamic landscape of CRC biomarkers and future, promising biomarkers involved in screening, diagnosis, and prognosis.

## 1. Introduction

In the current, fast-paced global environment, rectal cancer (RC) has become a central issue for health concerns. According to the Global Cancer Observatory: Cancer Today, RC ranked 8th in incidence, with a world age-standardized rate (ASR) of 7.1, and 10th in mortality, with a world ASR of 3.1, of all the cancers globally. Notably, the Asiatic continent is the leading region in incidence (57.2%) and mortality (59.2%), while in Europe, Hungary represents the country with the highest ASR incidence (17.2%) [1]. However, RC incidence has declined globally in recent years, especially after the year 2000, due to an improvement in the implementation of screening programs, with an annual decline in incidence for adults over 50 years of 1% per year. Of particular concern is an increase in incidence for adults under 50 years of 1–2% per year [2,3]. Due to changes in dietary habits and the growing consumption of fast food and processed foods, RC will exhibit great challenges in the near future. As studied by the US Preventive Services Task Force (USPSTF), nearly 10.5% of newly diagnosed colorectal cancer (CRC) cases are estimated to occur in individuals under the age of 50. Strikingly, the incidence of CRC, specifically adenocarcinoma, among adults aged between 40 and 49 has demonstrated a substantial increase of nearly 15% from the early twenties to 2014–2016 [3].

Therefore, the pursuit of early diagnosis in asymptomatic patients remains a main objective [4]. Liquid biopsy offers a new approach regarding the screening and diagnosis of CRC; it has become one of the great interests in solid tumors because of its capacity to identify a large spectrum of cancer-derived molecules from blood samples [5,6,7]. From these cancer-derived molecules, several represent a keen interest for researchers, such as circulating tumor cells (CTC) and circulating tumor DNA (ctDNA). These biomarkers have proved their ability to be used as indicators for minimal residual disease, to reduce the grade of invasiveness when searching for mutations, and to predict the radiological response [8,9]. Liquid biopsy, particularly in RC, holds promise for advanced applications such as monitoring responses to chemoradiation and evaluating the risk of disease recurrence. These applications are especially significant in the context of non-operative management strategies [6]. However, limited but promising data exist regarding the potential use of liquid biopsy for the early diagnosis of RC due to variable sensitivity that remains a challenge because of low concentrations of ctDNA. Regarding the prediction of response to chemoradiation, current data are inconclusive regarding the usefulness of pre-treatment liquid biopsy. Some studies suggest a positive correlation with dynamic monitoring (pre/post-treatment), but further research is needed to confirm these findings [10,11].

**Figure 1:**
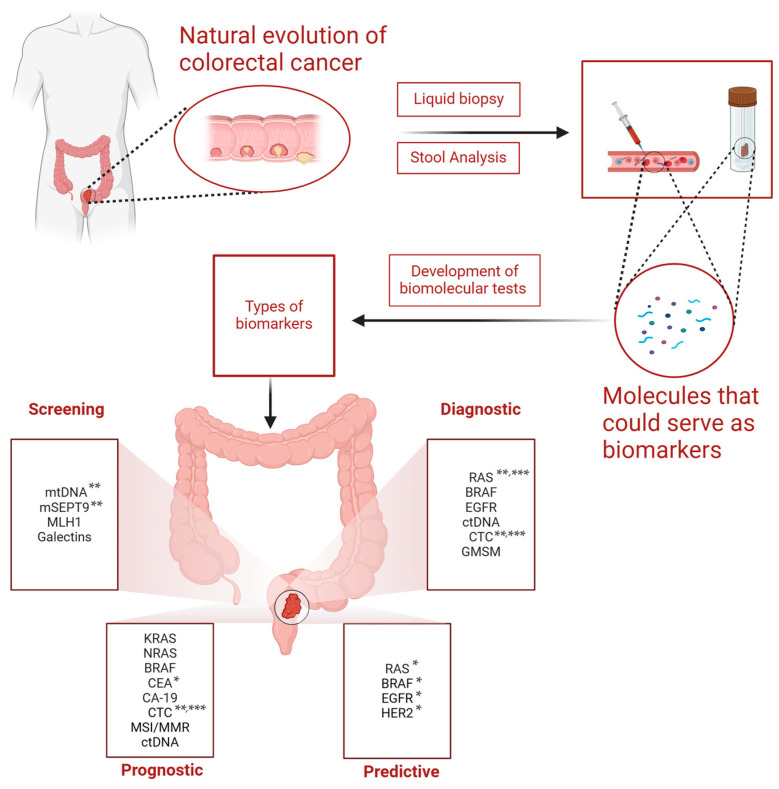
Actual and promising biomarkers used for screening, diagnosis, prognosis, and treatment prediction in colorectal cancer. The natural evolution of colorectal cancer involves dysplasia of normal cells, such as in adenomatous polyps, and metaplasia with the onset of cancer. At any stage, from premalignant lesions to cancerous lesions, liquid biopsy and stool analysis are used to evaluate molecules such as proteins, DNA, RNA fragments, circulating tumor cells, and other mutations. Instruments and tests are developed and, therefore, translated into clinical practice with the goal of establishing optimal biomarkers in the screening, diagnosis, prognosis, and prediction of treatment response. * Recommended by both NCCN and ESMO; ** FDA approved; *** EMA approved. Abbreviations: mtDNA, multi-target stool DNA; mSEPT9, methylated SEPTIN 9; MLH1, DNA mismatch repair protein Mlh1; RAS, rat sarcoma virus; BRAF, V-Raf Murine Sarcoma Viral Oncogene Homolog B; EGFR, Epidermal growth factor receptor; ctDNA, circulating tumor DNA; CTC, circulating tumor cell; GMSM, gut microbiome-associated serum metabolites; NRAS, neuroblastoma ras viral oncogene homolog; CEA, carcinoembryonic antigen; CA 19-9, carbohydrate antigen 19-9; MSI, microsatellite instability; MMR, DNA mismatch repair; HER2, receptor tyrosine-protein kinase erbB-2.

A biomarker is an objectively quantifiable paraclinical indicator assessed to serve as a sign of normal biological functions, pathological modifications, or pharmacological responses to therapeutic interventions [12]. However, while they may exhibit correlations, biomarkers do not inherently align with a patient’s subjective experience or overall sense of well-being [13]. The existing literature on RC extensively studies numerous molecular markers, reflecting the comprehensive nature of prior research (Figure 1). Given the substantial progress made thus far, our study aims to briefly concentrate on the contemporary landscape of biomarkers employed for screening, diagnosis, prognosis, and predictive response in RC. Additionally, our research will present promising biomarkers that, while not yet established in current practice, exhibit potential for future use in enhancing our understanding and management of RC.

## 2. Screening Biomarkers

Numerous blood-based markers have been suggested for CRC screening. However, only a few have been translated into clinical practice [14,15]. The transition from theoretical to established clinical utility remains a challenge, underscoring the complex process of validating and implementing biomarkers in routine medical settings [16]. Venugopal et al. emphasize the rising incidence of early-onset colorectal cancer (eoCRC). Numerous epidemiological studies have determined correlations between modifiable risk factors and their occurrence. However, despite these efforts, the cause responsible for the ascending trend in eoCRC incidence has yet to be determined [17,18]. The section below establishes the framework for established markers, such as stool DNA and SEPTIN 9, while presenting DNA mismatch repair proteins and galectins as developing biomarkers.

### 2.1. Multi-Target Stool DNA (sDNA)

At present, the sole biomarker employed in the detection and screening of early CRC in the United States is multi-targeted stool DNA testing, which includes a fecal immunochemical test (sDNA-FIT) as part of the component. However, the adoption of this relatively recent diagnostic tool remains limited, as it is perceived to be inferior to fecal immunochemical testing (FIT), which has found extensive utilization in both the United States and Europe for CRC screening. For example, Kamel et al. determined that the current UK screening program is based only on FIT, as well as in Germany [19]. The sDNA-FIT called Cologuard^®^ identifies the aberrant methylated N-myc downregulated gene 4 (NDRG4) and bone morphogenetic protein 3 (BMP3), Kirsten rat sarcoma virus (KRAS) mutations, and hemoglobin in stool samples. Imperiale et al. found that in 9989 patients, the sensitivity for detecting CRC was significantly higher with DNA testing (92.3%; 95% confidence interval CI, 83.0–97.5) compared to FIT (73.8%; 95% CI, 61.5–84.0), with a significant statistical difference of *p* = 0.002. In terms of specificity among participants with nonadvanced or negative findings, DNA testing demonstrated a specificity of 86.6% (95% CI, 85.9–87.2), while FIT exhibited a higher specificity of 94.9% (95% CI, 94.4–95.3), which was statistically significant with *p* < 0.001. The respective numbers of individuals who needed to be screened to detect a single cancer were 154 with colonoscopy, 166 with DNA testing, and 208 with FIT [20]. However, Ladabaum et al. emphasized that FIT and colonoscopy combined are more effective and cost-efficient compared to the sDNA-FIT when participation rates across patients are equal across all screening strategies [21]. In addition, a recent study by Vakil et al. reinforced the notion of high results of positive findings in sDNA-FIT but without findings in colonoscopy. Among the 1242 patients who tested positive, 226 individuals (18%) were subsequently diagnosed with either an advanced adenoma or CRC [22].

Nonetheless, a significant milestone was achieved in 2024 with the publication of the latest dataset regarding Cologuard Plus. The study of over 20,000 participants using next-generation multitarget stool DNA accomplished all the end-points of the study design, with a sensitivity of 94% (95% CI, 87.1–97.7), and a specificity for advanced neoplasia of 90.6% (95% CI, 90.1–91.0); while independent FIT scored 67.3% (95% CI 57.1–76.5) in sensitivity and 94.8% (95% CI 94.4–95.1) in terms of specificity for advanced neoplasia [23]. Therefore, the current status of sDNA enforces its superiority over traditional FIT, but additional rigorous studies are essential to evaluate the efficacy and diagnostic performance of sDNA compared to individual FIT; addressing the cost associated with sDNA-FIT is crucial for its widespread adoption.

### 2.2. Methylated SEPTIN 9 (mSEPT9)

Septins constitute a class of scaffolding proteins crucial for providing structural support during the process of cell division. Any abnormalities in the structure or expression of SEPT9 can consequently impact the intricate mechanism of cell division [24]. The mSEPT9 test (Epi procolon^®^, Epigenomics AG, Berlin, Germany) is a DNA serum test approved only in the United States. However, a meta-analysis carried out by Song et al. indicated that mSEPT9 assay superiority is present only in patients who were symptomatic when compared to FIT [25]. Additionally, Church et al., in their clinical trial, emphasized the reduced utility of SEPT9 as a screening tool. Its standardized sensitivity has been reported at 48.2 (95% CI, 32.4–63.6). Moreover, when stratified by CRC stages I–IV, the corresponding sensitivity values were 35.0% (95% CI, 13.3–59.6) for stage I, 63.0% (95% CI, 32.5–87.7) for stage II, 46.0% (95% CI, 32.5–87.7) for stage III (95% CI, 16.5–85.4), and 77.4% (95% CI, 23.7–100) for stage IV. These findings highlight the variable performance of the test across different stages of CRC, with higher sensitivity observed in the advanced stages (stages III and IV) compared to the earlier stages (stages I and II) [26]. Furthermore, Nian et al. showed in their meta-analysis that the positive ratio of methylated mSEPT9 was found to be higher in advanced CRC stages, with percentages increasing from 45% (95% CI, 0.38–0.53) in stage I to 79% in stage IV (95% CI, 0.69–0.87) (specifically, 70% [95% CI, 0.60–0.79] in stage II) and 76% [95% CI, 0.64–0.86] in stage III). There was an observable correlation with the degree of tissue differentiation, where the positive ratio was 31% (95% CI, 0.12–0.59) in good differentiation, 73% (95% CI, 0.68–0.78) in moderate differentiation, and 90% (95% CI, 0.83–0.95) in poor differentiation tissue [27]. On the other hand, in a recent study by Lu et al., they achieved promising results. In their study, the application of mSEPT9 as a diagnostic tool demonstrated an overall sensitivity of 72.94% (95% CI, upper limit 79.15%) and a specificity of 81.97% (95% CI, lower limit 86%). Notably, when combining mSEPT9 with traditional biomarkers such as carcinoembryonic antigen (CEA) and carbohydrate antigen 19-9 (CA 19-9), there was an enhancement in diagnostic performance. The combined approach yielded an improved sensitivity of 78.43% (95% CI, upper limit 84.89%) and a specificity of 86.07% (95% CI, lower limit 90.62%) [28].

As discussed above, the mSEPT9 exhibits variability regarding its sensitivity and specificity. Sensitivity values range in the literature from 50.9% to 93.1%, while specificity values range from 62.2% to 93.8%. This variability underscores the importance of establishing strict testing protocols, as well as the need for standardized methodologies to ensure consistency and reliability [28]. The emergence of mSEPT9 DNA serum testing represents a potential addition to the screening arsenal for CRC. However, it currently stands as a third-line alternative for patients who express a reluctance to engage in traditional screening methods. Despite its potential, the adoption of mSEPT9 testing is constrained by the ongoing quest for enhanced accuracy, necessitating further refinement before it can be widely embraced as a first-line screening option [19].

### 2.3. DNA Mismatch Repair Protein

Lynch syndrome (LS) is a well-known factor that makes the patient susceptible to CRC. In conjunction with studies elucidating the role of aberrant DNA methylation in the pathogenesis of CRC, research conducted by Early Detection Research Network (EDRN) Principal Investigators (PIs) has illustrated the viability of serum methylated MLH1 as a blood-based biomarker for CRC detection. Furthermore, they have shown that aberrantly methylated genes can be employed as markers for the detection of colon adenomas and CRC when utilizing stool DNA-based approaches. This suggests the potential utility of DNA methylation patterns as clinically relevant biomarkers in non-invasive screening methodologies for colorectal neoplasms [14]. Cenin et al. emphasized that MutL homolog 1 (MLH1) gene detection is more cost-effective compared to the V-Raf Murine Sarcoma Viral Oncogene Homolog B (BRAF) pathway across all age-at-diagnosis thresholds. Using a universal approach of screening using the MLH1 pathway instead of the BRAF pathway would reduce the annual point estimate cost by 6292 Australian dollars [29]. However, Helderman et al. highlighted that recent research indicates that MLH1 promoter methylation (MLH1-PM) and pathogenic constitutional mismatch repair variants are not necessarily mutually exclusive. This growing evidence underscores the complexity of LS diagnostics, suggesting that the presence of MLH1-PM should not automatically lead to the exclusion of a diagnosis of LS [30].

### 2.4. The Galectins Family

Galectins, a family of carbohydrate-binding proteins with a distinct affinity for β-galactosides, are widely distributed and evolutionarily conserved. Among them, Galectin-3 has been implicated in various forms of cancer, playing a functional role in progression and metastasis. Notably, elevated levels of Galectin-3 are observed in the blood of individuals with CRC. Ongoing evaluations of the Galectin-3 ligand have shown promising results. For example, Bresalier et al. displayed that the test has a sensitivity of 70% for early-stage cancer and 88% for late-stage cancer while maintaining a specificity of 90% and an area under curve (AUC) from 0.87 to 0.90. Importantly, all models adhered to predefined criteria for the detection of advanced adenoma and screen-relevant neoplasia, encompassing both cancer and advanced adenoma cases [14]. Moreover, Nikolau et al. showed in their meta-analysis that Galectin-4 (LGALS4) alone had a sensitivity of 82.1%, a specificity of 61.2%, and an AUC of 0.746; when combined with Tetraspanin 8 (TSPAN8), the sensitivity raised to 92.54%, with a specificity of up to 67.16% and an AUC of up to 0.862 [31].

Beyond currently approved screening tests, ongoing developments in CRC screening include the exploration of novel markers such as fecal- and blood-based microRNA, as well as markers associated with the CRC-related gut microbiome [32]. Moreover, tools that assess circulating tumor DNA (ctDNA) [33], the circulating tumor cell (CTC) [34], microRNA (miRNA) [35], circular RNA (circRNA) [36], and nucleosomes [37] are in the process of being developed as screening biomarkers.

Despite the advances made in terms of the technology used to improve the sensitivity of biomarkers and to develop new ones, these results and national screening programs cannot fully screen each person. Therefore, tumoral cells can overgrow, and cancer then develops. The next chapter moves on to set out the framework for the diagnostic biomarkers involved in CRC.

## 3. Diagnostic Biomarkers

### 3.1. RAS, BRAF, and EGFR Mutations

Among new CRC diagnoses, 20% of patients present with metastatic disease at the time of diagnosis [38]. Currently, several biomarker assays are employed for the diagnosis of CRC, specifically focusing on Rat sarcoma virus (RAS) genes, rapidly accelerated fibrosarcoma (RAF) genes, and epidermal growth factor receptor (EGFR) genes. Notable among these are OncoBEAM, Idylla, AdnaTest ColonCancerSelect, and AdnaTest ColonCancerDetect [39]. Vidal et al. supported the use of OncoBEAM as a diagnostic biomarker. The overall likelihood of RAS results between the ctDNA RAS OncoBEAM assay and standard techniques for tissue analysis was notably high, reaching 93% agreement in a cohort of 115 patients, with a kappa index of 0.844 (95% CI, 0.746–0.941) [40]. Regarding the Idylla test, Zekri et al., in their study on 64 samples evaluating exons 2, 3, and 4, showed that the identification rate for KRAS was reported at 93.3%, pointing to a high level of agreement between conventional testing methods for this specific gene. Similarly, for the neuroblastoma ras viral oncogene homolog (NRAS), the accuracy rate was notably high, at 94.4%; for mutated and wild-type (WD) variants, a concordance of 93.8% was achieved [41]. Additionally, the study by Bettegowda et al. on 206 metastatic CRCs revealed that ctDNA exhibited a sensitivity of 87.2% in detecting clinically relevant KRAS gene mutations, while the specificity of ctDNA was reported at 99.2%. The concordance between plasma levels and tumor tissue was 95% and was statistically significant (k factor = 0.88, *p* < 0.0001) [42]. However, the current utilization of molecular biomarkers in the detection of CRC is restricted. For example, in the UK, the singular serum biomarker for CRC that undergoes testing is the CEA [43].

### 3.2. ctDNA

The majority of cell-free DNA in plasma originates from leukocytes, yet malignancy can release detectable amounts of DNA into the circulation. Tests that utilize alterations in ctDNA exhibit high specificity. However, when employed as standalone tests, these assays may encounter limitations in sensitivity. The CancerSEEK—a ctDNA-protein test—demonstrated promising results in 812 individuals, showcasing a sensitivity of approximately 65% (95% CI, 60–70) for CRC. Notably, the specificity of CancerSEEK surpassed 99%. Worth mentioning is the merging of CancerSeek with machine learning software to predict the origin of the primary tumor and to establish the most appropriate approach; in CRC, the accuracy of the prediction was between 84–100% [44]. Moreover, Bessa et al. presented a newly developed multimodal blood-based test with a sensitivity of 93% in 323 samples for detecting CRC at a specificity of 90% in 264 samples. Specifically, the sensitivity was 84% out of 49 samples (stage I), 94% out of 196 samples (stage II), and 96% out of 73 samples (stage III). Sensitivity to identify advanced precancerous lesions initially stood at 14%, with sensitivity improved to 23% after some refinements, but with a decrease in specificity down to 86% [45]. Therefore, Hanna et al. emphasized that the evolution of modern next-generation sequencing (NGS) and advanced polymerase chain reaction (PCR) technologies, coupled with the integration of machine learning and artificial intelligence, has paved the way for innovative cancer biomarker panels such as DNA mutations, methylation, and fragmentomics, collectively forming a comprehensive set of markers. This synergy has given rise to ctDNA detection assays with the potential for the sensitive and specific identification of early-stage cancers and even precancerous conditions [46].

### 3.3. CTC

Currently, the gold standard for the diagnosis of CRC involves a dual approach, incorporating both colonoscopy and subsequent histopathological examinations. On the other hand, CTCs are epithelial cancer cells that can be identified within the bloodstream [47]. However, the isolation of CTCs poses challenges due to their rarity among hematological cells, their short ex vivo half-life, and the absence of a single universal CTC-specific marker. In response to these limitations, CTC enrichment through immunoaffinity has emerged as the prevailing strategy for their isolation [48]. Therefore, Allard et al., in their work on 333 specimens from 196 metastatic CRC, determined the presence of at least 2 CTCs in 30% of the specimens from patients diagnosed with CRC (17% for 5 or more CTCs, 9% for 10 or more CTCs, 2% for 50 or more CTCs) [49]. Moreover, Bahnassy et al. proposed an additional approach. They combined several different techniques simultaneously—Flowcytometry (FCM), CellSearch (CS), cytokeratin 19 (CK19), mucin1 (MUC1), cluster of differentiation (CD) 44, CD133, and aldehyde dehydrogenase 1 (ALD1H)—developing a multiplex test for the detection of at least four CTC/7.5 mL. Therefore, when the multiplex approach was tested on 63 non-metastatic RC patients, the sensitivity rate was reported to be 68.3% at 95% specificity and an accuracy of 83.2%. In comparison, the CellSearch method alone yielded a lower sensitivity rate of 54% at 95% specificity and an accuracy of 76.9% [50]. However, the variability in reported CTC numbers across different platforms underscores the imperative for standardization in defining and characterizing CTCs. This necessitates the establishment of uniform definitions and clear criteria for identifying and categorizing objects as CTCs. The lack of standardized guidelines can lead to discrepancies in reported CTC counts, hindering the comparability and reliability of findings across studies and technologies [51].

### 3.4. Gut Microbiome-Associated Serum Metabolites (GMSMs)

**Figure 2:**
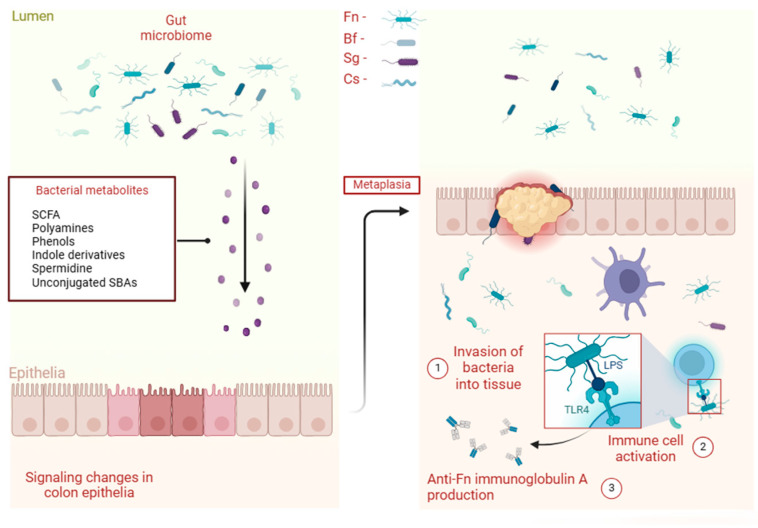
Types of bacteria and their metabolites involved in colorectal cancer. Bacteria such as Fn, Bf, Sg, and Cs produce metabolites during carbohydrate metabolism, such as short-chain fatty acids (SCFA), protein metabolism (polyamines, phenols, indole derivatives, and spermidine), and lipid metabolism (unconjugated SBAs), that promote molecular changes in the gut epithelium. Following prolonged inflammation, metaplasia occurs, and therefore bacteria penetrate the tumor cell as well as the colorectal epithelium. Immune activation begins, and LPS—found on the surface of bacteria such as Fn—stimulates TLR4 to produce further anti-immunoglobulin A that could be found in the bloodstream. Abbreviations: Fn, Fusobacterium nucleatum; Bf, Bacteroides fragilis; Sg, Streptococcus gallolyticus; Cs, Clostridium septicum; SCFA, short-chain fatty acids; SBAs, secondary bile acids; LPS, lipopolysaccharide; TLR4, toll-like receptor 4.

The gut microbiota exhibits a close association with the onset and development of human cancers due to the entering of metabolites—generated by gut bacteria—in the systemic circulation and undertaking regulatory roles (Figure 2). As a result, Chen et al. defined a panel with 8 GMSMs evaluated on a cohort of 156 patients. Their model achieved an AUC of 0.92 with a sensitivity of 83.5% and a specificity of 84.9%. While stratified by the type of tumor, adenoma scored an AUC of 0.84, a sensitivity of 63.2%, and a specificity of 84.9%; stage I and II CRC achieved an AUC of 0.93 with a sensitivity of 88.2% and a specificity of 84.9%; stage III/IV CRC attained an AUC of 0.91, a sensitivity of 84.2%, and a specificity of 84.9% [52]. Kwong et al. supported this evidence in their study on 13,096 positive blood culture tests. They found that bacteremia with Bacteroides fragilis (Bf) (hazard ratio [HR] = 3.85; 95% CI, 2.62–5.64), Streptococcus gallolyticus (Sg) (HR = 5.73; 95% CI, 2.18–15.1), Fusobacterium nucleatum (Fn) (HR = 6.89; 95% CI, 1.70–27.9), and Clostridium septicum (Cs) (HR = 17.1; 95% CI, 1.82–160), and other previously known CRC-associated bacteria, were statistically significant as a factor that increased the risk of CRC [53]. Moreover, Wang et al. found that Fn infection triggered a significant elevation in serum anti-Fn antibodies among CRC patients. Notably, the serum anti-Fn immunoglobulin A (IgA) level emerged as a potential diagnostic biomarker for CRC. When combined with CEA and CA 19-9, the inclusion of serum anti-Fn-IgA contributed to increased sensitivity (40%) in the detection of early-stage colorectal cancer with a specificity of 94.22%, an AUC of 0.74, and a positive predictive value (PPV) of 56.4% [54]. Alhhazmi et al. showed that in CRC, two microbial markers, Fusobacterium spp. and Porphyromonas, exhibited elevated levels compared to the healthy controls. However, the profiling of metabolite markers in CRC versus the healthy controls across seven studies yielded conflicting results, with no consensus on common markers identified among them [55].

Nonetheless, significant work was carried out in early 2024 on the oral microbiome. Zepeda-Rivera et al. published their results on Fn. Through large-scale culturing, sequencing, and comparative genomic analyses, they found that Fn correlated with distinct CRC-enriched genetic factors. Specifically, a clade of Fn animalis—called Fna C2—dominates the human CRC tumor niche by altering intestinal metabolism and increasing oxidative stress in an animal CRC model. Therefore, with these notable results and due to its high status of virulency, Fna C2 could become a target for inhibition and study on pathogenicity in CRC [56].

A study carried out by Nikolaou et al. found several encouraging diagnostic biomarkers: interleukin-8, long non-coding RNA, DNA methylation, SEPT9, CA 11-19, and a membrane protein. All these biomarkers showed a sensitivity of over 70% and a specificity of over 90% [31]. Moreover, Luo et al. and Li et al. focused their attention on viable biomarkers in several types of RNA, such as non-coding RNA, lncRNA, miRNA, and circRNA [57,58]. The existing diagnostic biomarkers for CRC necessitate further standardization before widespread adoption. They are often difficult to assess, costly, and potentially hazardous. Moreover, not all studies have thoroughly evaluated their markers’ efficacy in detecting early-stage CRC, variability in cutoff values, the methodology used for marker analysis, and the timing of sample collection, thus contributing significantly to result heterogeneity. Additionally, the lack of specificity regarding the exact region of the colon or rectum assessed in these studies adds a layer of complexity to the interpretation of results.

This section analyzed actual established diagnostic biomarkers (RAS, BRAF, EGFR gene mutations, and ctDNA) while presenting promising elements such as CTC and molecules derived from gut bacteria. The next part of this paper will establish the framework for grounded prognostic tools—for instance, CEA and CA 19-9—and novel biomarkers such as CTC, KRAS/NRAS/BRAF, MSI/MMR, and ctDNA.

## 4. Prognostic Biomarkers

### 4.1. CEA, CA 19-9

CEA, a glycoprotein, is a serum biomarker that shows elevated levels not only in CRC but also in various other malignancies. On the other hand, briefly, CA 19-9 is a cell surface glycoprotein complex. Currently, according to the National Comprehensive Cancer Network (NCCN), tumoral biomarkers such as CEA and CA 19-9 can be examined and utilized as prognostic indicators in patients undergoing cytoreductive surgery and hyperthermic intraperitoneal chemotherapy [59]. Therefore, NCCN concluded that an elevated preoperative level of CEA and CA 19-9 was associated with unfavorable progression-free survival (PFS). Additionally, vigilant surveillance is crucial for individuals contemplating a watch-and-wait approach to promptly address potential tumor regrowth. Hence, CEA assessments are recommended every 4 months during the initial 2 years, followed by a transition to every 6 months from years 3 to 5 [60]. Moreover, Campos-da-Paz et al. exposed that contemporary research is centered on utilizing CEA as a target in various domains, such as drug delivery systems, photodynamic therapy, radioimmunotherapy, cancer imaging, and nanotechnological devices [61]. This has resulted in numerous patents related to the development of anti-CEA antibodies or their fragments, showing promise in targeting CRC and liver metastasis cells [62]. However, Björkman et al. proposed that cancer antigen 125 (CA 125) could stand out as a substantial and independent prognostic factor in CRC patients, exhibiting superiority over CEA. Additionally, they proposed carbohydrate antigen 242 (CA 242) as a more effective prognostic marker compared to both CEA and CA 19-9. In their study of 148 patients, high levels of CEA were correlated with a poor prognosis (HR 2.32; 95% CI, 1.56–3.45), CA 125 (HR 2.48; 95% CI, 1.68–3.65), as well as CA 242 (HR 3.23; 95% CI, 2.13–4.91), with results being statistically significant. Moreover, 5-year survival was 58% (95% CI, 50–66%) at high CEA levels, 53% (95% CI, 42–64%) at elevated CA 125 levels, 55% (95% CI, 47–63%) at high levels of CA 242, and 53% (95% CI, 44–62%) in patients with high CA 19-9. Worth mentioning is the survival specific to RC, which was the lowest in patients with high levels of CA 242 (HR = 3,4; 95% CI, 1.94–5.79) compared to high levels of CA 125 (HR = 2,1; 95% CI, 1.14–3.69) [63].

### 4.2. CTC

As previously mentioned, CTCs are capable of moving, migrating, and invading blood vessels and could represent a main mechanism in metastasis. Magri et al. emphasized their prognostic role. They focused on CTCs as a marker for early-stage metastasis. Therefore, CTCs could provide a means to correlate their quantity with prognosis. Additionally, the successive analysis of CTCs facilitates the monitoring of cancer evolution throughout the treatment trajectory, enabling the early detection of drug resistance and the assessment of anticancer drug efficacy [64]. However, Vasseur et al. identified two studies in their review that offer contradictory evidence [65]. On the one hand, one study showed that preoperative CTC detection using CellSearch^®^ (Menarini Silicon Biosystems, Bologna, Italy), employing a cutoff of at least 1 CTC, emerged as an independent prognostic marker, while another study reported no association post-surgery in a cohort of 519 patients using at least 1 CTC/7.5 mL cutoff. Nonetheless, Yu et al. observed that cell surface vimentin-circulating tumor cells (CSV-CTCs) dominate among CTCs in CRC patients. Moreover, a count of CSV-CTCs ≥3 has been identified as an independent risk factor associated with a poor prognosis (HR  =  3.78, 95% CI, 1.55–9.26; p  =  0.04). Presently, there is limited understanding regarding the phenotypes of CTCs, specifically epithelial and mesenchymal phenotypes, and their implications in the prognosis of CRC [66].

### 4.3. KRAS/NRAS/BRAF

Currently, the Intplex test stands as the sole approved prognostic test in CRC, specifically designed to identify KRAS/NRAS/BRAF point mutations in plasma [39]. NCCN guidelines recommend that all patients with metastatic disease should undergo RAS and BRAF mutation analysis, with testing being performed from the primary or metastatic site in order to establish an appropriate treatment decision. In the context of prognosis, a study by Perdyan et al. summarized that the presence or elevated concentrations of KRAS mutations in plasma or serum were correlated with poor prognosis in terms of overall survival (OS), PFS, and disease-free survival (DFS). Therefore, the determination of specific cut-off concentrations is deemed necessary for the continued clinical implementation of liquid biopsy in CRC [67]. Moreover, Ogunwobi et al. exposed that the presence of the BRAF mutation is linked to poor survival outcomes, encompassing both reduced PFS and up to 50% worse OS when compared to patients with the BRAF wildtype [68].

### 4.4. MSI/MMR

CRC is characterized by MSI arising in different percentages according to the status of the cancer. In the case of non-metastatic malignancy, CRC due to MMR represents 15%, while in metastatic disease, the proportion is around 5%. The prognostic significance of MSI status in non-metastatic CRCs has been extensively investigated. However, numerous NCCN member institutions and other comprehensive cancer centers recommend conducting MSI testing. Its recommendation is maintained also in a family with no history of hereditary cancer. This approach is undertaken to identify patients who should undergo genetic testing for LS. Moreover, the cost-effectiveness of the test, specifically for CRC, has been validated, and this strategy has garnered endorsement from relevant working groups [60]. Additionally, Zhang et al. described that patients with MSI have different prognoses based on cancer staging. For stage II CRC, patients exhibit a 5-year survival rate as high as 80%. Regarding stage III, patients who received postoperative adjuvant chemotherapy had better outcomes than microsatellite stability patients. In the context of stage IV, they pointed out that in four clinical studies, the median OS was reported as 13.6 months for patients with deficient DNA mismatch repair (dMMR) and 16.8 months for patients with proficient DNA mismatch repair (pMMR) [69]. Moreover, the meta-analysis of Toh et al. revealed that high microsatellite instability (MSI-H) CRC demonstrated an overall survival benefit with a lower rate of dissemination. The survival advantage was prominently observed in both stage II and III CRC, but the MSI-H did not prove to be a robust prognostic marker in stage I or stage IV CRC without immunotherapy [70]. However, in a recent meta-analysis by Wang et al., they emphasized that for stage III CRC, MSI-H did not exhibit a prognostic impact on OS, DFS, and disease-specific survival (DSS) [71].

### 4.5. ctDNA

ctDNA has started to capture the attention of researchers as a potential prognostic biomarker. For example, Kotani et al., in their cohort study with 1039 patients, examined the existence of ctDNA after surgery. They measured the level of ctDNA at 4 weeks after surgery, with 187 patients being tested for positive results. Following the results, they observed that patients who exhibited ctDNA had a ten times higher risk of recurrence than ctDNA-negative patients (HR = 10; 95% CI, 7.7–14, *p* < 0.0001); DFS was 38.4% (95% CI; 31.4–45.5%) in ctDNA-present patients, while in ctDNA-absent patients, DFS was 90.5% (95% CI 88.3–92.3%). Additionally, they observed its prognostic role according to the staging of CRC. Patients who had been diagnosed, using a multivariate analysis, with stage II or III CRC and had positive values of ctDNA had 11 times (HR = 10.82; 95% CI, 7.07–16.6, *p*  < 0.001) the chance of recurrence in comparison to patients without ctDNA [72]. Moreover, Dasari et al. determined that the presence of ctDNA after curative surgery may show a residual disease. As a result, ctDNA may have good sensitivity and specificity in predicting the recurrence of the disease [73]. Additionally, Tie et al. evaluated 1046 plasma samples from 230 patients the role of ctDNA in stage II CRC patients. Overall, 7.9% of 178 patients not treated with chemotherapy but with ctDNA had a recurrence diagnosed at a median of 27 months. Moreover, the presence of ctDNA statistically significantly diminished the recurrence-free survival (RFS) (HR = 18; 95% CI, 7.9–40) [74]. Furthermore, a recent meta-analysis by Faulkner et al. involving 3002 patients supported the previous results that showed a poorer PFS if ctDNA is present. At the initial liquid biopsy post-surgery, patients with ctDNA had seven times the risk of death in contrast to ctDNA-free patients (HR = 6.92; 95% CI, 4.49–10.64) [75].

The section above analyzed the framework for prognostic CRC biomarkers, focusing on traditional markers such as CEA and CA 19-9, along with novel biomarkers such as CTCs, KRAS/NRAS/BRAF mutations, MSI/MMR, and ctDNA. Furthermore, the next chapter transitions to predictive biomarkers, including RAS/BRAF/EGFR mutations, MSI/MMR, and receptor tyrosine-protein kinase erbB-2 (HER2), which guides therapeutic decision-making in CRC management.

## 5. Predictive Biomarkers

### 5.1. RAS/BRAF/EGFR Mutations

A predictive biomarker is an indicator employed in customizing treatments on an individual basis, aligning with the molecular subtype of the cancer. With the growing prominence of targeted therapy in the treatment of advanced or metastatic CRC, the NCCN Panel recommends the determination of tumor gene status for KRAS/NRAS and BRAF mutations [60]. Therefore, the mutations in these genes are acknowledged by the American Society of Clinical Oncology (ASCO), the European Society for Medical Oncology (ESSMO), and NCCN as an important predictive biomarker [24]; however, the specific therapy following the type of mutation is a subject that is not the main focus of this article. Ogunwobi et al. exposed that KRAS mutations are linked to a poor response to anti-EGFR receptor therapy, while in KRAS wildtype patients, there was a notable 16% increase in the overall response rate due to therapy. Given that KRAS mutations are present in up to 40% of patients, identifying this subset allows for the avoidance of expensive anti-EGFR treatment in a significant percentage of the patient population [68]. Mattia et al., in their meta-analysis, summarized that the presence of a KRAS mutation plays a significant detrimental role and has been identified as a predictive factor of a poor response to neoadjuvant treatment in patients with locally advanced rectal cancer (LARC). In wild-type KRAS patients, an improvement of 20% in comparison to mutated KRAS patients in terms of pathological complete response (pCR) was achieved when using cetuximab (95% CI, 14–29%). When assessed for heterogeneity, mutated KRAS was associated with a decrease of almost two times (OR = 1.80; 95% CI, 1.23–2.64) in pCR. Worth mentioning is that when stratified by the use of cetuximab or not, the use of cetuximab achieved a reduction by almost 2.5 times in tumor progression (OR = 0.89; 95% CI 0.39–2.05); no cetuximab patients (OR = 2.17; 95% CI 1.41–3.33). Moreover, they exposed the limitations of these biomarkers in RC, where their role is not very well known [76]. For example, preclinical investigations have suggested that KRAS mutations could promote a more aggressive tumor phenotype and also confer resistance to radiotherapy [77,78].

### 5.2. MSI/MMR

As previously mentioned, MSI is characterized by tumors possessing a defective DNA mismatch repair system, primarily resulting from the inactivation of specific genes. Actual guidelines by NCCN emphasize the crucial significance of determining MSI and MMR status at the time of diagnosis because treatment recommendations for RC can vary significantly across all stages, depending on the results of these biomarkers. Despite a scarcity of data in this context, the guideline recommends pembrolizumab or nivolumab, either as monotherapy or in combination with ipilimumab, as potential options for neoadjuvant therapy in dMMR/MSI-H metastatic colorectal cancer (mCRC) [79]. Although clinical trial and cohort studies data supporting this approach are lacking, a few case studies have reported significant responses to pembrolizumab and nivolumab when employed as neoadjuvant therapy for advanced or metastatic dMMR [60,80]. However, Ooki et al. identified a clinical trial in their work in which pembrolizumab has been shown to have a superior PFS; 16.5 months in comparison to 8.2 months in classic chemotherapy (HR = 0.60; 95% CI, 0.45–0.80; *p* = 0.0002) [77]. However, while the Food and Drug Administration (FDA) granted approval for pembrolizumab as a first-line treatment for patients with dMMR/MSI-H mCRC in June 2020, neither the Japanese Pharmaceuticals and Medical Devices Agency nor the European Medicines Agency have endorsed pembrolizumab for use as a frontline regimen in this context. Moreover, Taieb et al. highlighted that despite the efficacy of immune checkpoint inhibitors (ICIs), both primary and secondary resistance are observed in over 50% of patients with MSI-H/dMMR CRC. Therefore, identifying these patients and developing strategies to overcome resistance will pose a significant challenge [81].

### 5.3. Receptor Tyrosine-Protein Kinase Erbb-2 (HER2)

HER2 is a protein that normally resides in the membranes of cells and is encoded by the erythroblastic oncogene B (ERBB2) gene. In CRC, HER2 overexpression and amplification have been explored as potential therapeutic targets. Moreover, HER2 overexpression has been associated with resistance to anti-EGFR therapy. Dong et al. reported that incidence rates of HER2 overexpression or amplification in CRC exhibit considerable variability, ranging from 0% to 83% [82]. NCCN guidelines recommend, in addition to the testing of KRAS/NRAS and BRAF and MSI/MMR status, as previously mentioned, the testing of HER2 amplifications. However, HER2 testing is not indicated if the tumor is already known to have a KRAS/NRAS or BRAF mutation. Anti-HER2 therapy is specifically indicated in tumors that exhibit HER2 amplification and are concurrently RAS and BRAF wild-type. Additionally, beyond its role as a predictive marker for HER2-targeted therapy, it has shown initial indications of being predictive of resistance to EGFR-targeting monoclonal antibodies. However, the work included a cohort of 98 patients [60]. In the evolving domain of radiogenomics, the integration of radiological and genetic features may offer enhanced prognostic sensitivity compared to either modality alone [83,84]. The imperative to explore additional biomarkers is underscored by the current recommendation in national guidelines, which primarily includes KRAS, NRAS, BRAF, and MSI status for evaluating treatment response and predicting outcomes in CRC [85].

A table summarizing each biomarker category (screening, diagnostic, predictive, and prognostic) along with a brief methodological detail, specificity, and sensitivity is presented in Table 1.

## 6. Conclusions and Future Directions

The purpose of the current study was to critically appraise the recent advancements in molecular screening, diagnosis, and prognosis of RC. One of the most significant findings to emerge from our study is the increased interest in the development of novel biomarkers such as ctDNA and CTC. While FIT remains widely utilized globally, the newest results of Cologuard Plus show major improvements in biomolecular testing with the aid of NGS; mSEPT9 exhibits promising potential as a biomarker, although further studies are warranted to ascertain its superiority; MLH-1 is being developed for the detection of colon adenomas and CRC through stool-based DNA testing, while galectins are also under investigation for CRC screening. Therefore, a stepwise integration of these various biomarkers can significantly enhance the precision of the screening and diagnosis of eoCRC. Initially, widely used tests such as the FIT and Cologuard Plus could be deployed for preliminary screening, detecting early CRC markers from stool samples; this stage benefits from recent biomolecular advancements, particularly in next-generation sequencing NGS. For individuals with positive results, mSEPT9 could be introduced to add specificity by detecting epigenetic alterations, strengthening early detection accuracy. Subsequently, in patients at elevated risk or those with confirmed disease, MLH-1 stool-based DNA tests can assess the presence of adenomas or early CRC, improving risk stratification and therapeutic options. Lastly, galectins, currently under investigation, may eventually be incorporated to refine early detection, ensuring a comprehensive approach to screening. This stepwise biomarker combination optimizes patient stratification and could lead to improved clinical outcomes in CRC management.

In the context of diagnosis, the BRAF pathway serves as a frequently employed biomarker; however, liquid biopsy techniques that imply the analysis of ctDNA and CTCs offer promising results. Additionally, the investigation of GMSM shows potential for reshaping CRC screening, diagnosis, and prognosis, especially with the aid of the latest results on Fn. Regarding prognosis, while CEA and CA 19-9 are commonly used, CTCs, KRAS/NRAS/BRAF mutations, MSI, and ctDNA exhibit promising prognostic value. Furthermore, predictive biomarkers such as RAS/BRAF/EGFR mutations, MSI/MMR status, and HER2 status are utilized to assess the response to immunotherapy.

However, the biomarkers discussed present several limitations. Regarding screening tests, the existing work fails to resolve the contradiction between sDNA and FIT prices; for example, a FIT test price varies between $31.99 and $89, while the Cologuard price is estimated to reach a maximum of $649 [88]. Another limitation of sDNA, particularly Cologuard Plus, is that the performance was not directly evaluated in comparison with the current sDNA test (Cologuard) [23]. On the same note, MSI/MMR proteins significantly raise the price of screening at all age categories, therefore making it a non-feasible biomarker in the actual economic context. Previous studies concerning mSEPT9 have not dealt with the variability results that follow this test. Such expositions are unsatisfactory because values ranging from 50–93% (sensitivity) could drastically impact the number of patients successfully screened, which is of great interest in the preventive era on which physicians and governments should focus. Observing the Galectins family, large gaps in terms of sensitivity and specificity across different subtypes of galectins make them a limited screening tool. In addition, the lack of data about populations, as well as variations in genetic backgrounds and environmental factors, limits their value. In terms of diagnostic biomarkers, OncoBEAM and Idylla also show variability in sensibility and specificity; CancerSEEK demonstrates limited sensitivity for detecting early-stage CRC and precancerous lesions; ctDNA detection and CTCs offer promising results with the aid of liquid biopsy, but their application in routine clinical practice remains limited due to challenges in sensitivity, specificity, cost, and the need for specialized equipment and expertise. Moreover, the lack of consistency limits the comparability of results between studies. In the era of gut studies, microbial markers like Fusobacterium nucleatum have been associated with CRC, although studies show conflicting results regarding their diagnostic utility, and the lack of standardization in cutoff values and methodologies used for biomarker analysis restricts their actual use. Involving prognostic biomarkers, CEA and CA 19-9 remain the most used due to their feasibility. CTCs lack results standardization (cutoff values of 1 CTC vs. ≥3 CTCs), limited understanding of the diverse phenotypes of CTCs, such as epithelial and mesenchymal types; in early-stage cancers, ctDNA levels may be too low for reliable detection; great differences in methodologies, sample sizes, and cutoff values across studies result in reduced reliability, and therefore large clinical trials should be addressed.

These findings suggest that, in the current era, with the focus on targeted therapy and quality of life, biomarkers identified using liquid biopsy or stool analysis dominate the interests of researchers [89]. The rationale lies in the screening and diagnostics used to minimize the patient’s discomfort. Not only ctDNA and CTCs are of interest, but as previously mentioned, work on different types of RNA—such as microRNA and circRNA—or nucleosomes is studied to evaluate their feasibility as screening and diagnostic biomarkers. Therefore, intensive support for such novelties is needed. Firstly, physicians should discuss with their patients the new techniques to increase the number of cohorts for further studies. Secondly, large assemblies should define and construct rigorous protocols for the development of large clinical trials to accurately evaluate their sensitivity, specificity, and accuracy across different tests, populations, genetic variations, and healthcare systems [90]. Thirdly, both governments and non-governmental entities should emphasize the role of screening in a population through solid and innovative screening programs, which may reduce the burden of disease and the pressure on the healthcare system, allowing physicians and researchers to further improve these tests [91,92].

Moreover, with the aid of machine learning (ML) and more recently artificial intelligence (AI), these biomolecular tests may show an improvement in their accuracy. This can be achieved by enhancing the predictive accuracy and personalization of treatment strategies. One key milestone is the use of AI to analyze large, complex datasets from multi-omics platforms, such as genomics, transcriptomics, and proteomics, to identify novel biomarker signatures. This approach could accelerate the support biomarkers such as ctDNA, mSEPT9, and CTCs, improving their sensitivity and specificity. Moreover, AI algorithms could also aid in integrating various biomarkers, such as MLH-1 and galectins, across different stages of CRC, optimizing their use in screening, diagnosis, and prognosis. Another milestone involves the application of ML in the real-time analysis of ctDNA and CTCs, allowing for more accurate monitoring of disease progression and the early detection of recurrence. Furthermore, AI-driven predictive models can facilitate individualized treatment by correlating specific biomarker profiles with clinical outcomes, enhancing treatment response predictions. Over the coming years, AI will likely play a crucial role in validating emerging biomarkers, refining multi-biomarker approaches, and personalizing CRC management, ultimately improving patient outcomes and optimizing therapeutic decision-making.

The study contributes to our understanding of the vast field of biomolecular testing, with numerous and exhaustively potential biomarkers that could be translated into the clinical field. Future studies would benefit from standardized and more rigorous protocols to evaluate the screening, diagnostic, prognostic, and predictive values of some biomarkers, while further studies need to be carried out in order to validate the results of not widely acknowledged markers. Moreover, analysis using non-classical tests implies an imbalanced cost-efficacy. Therefore, further research should be oriented to reduce the cost of tests—especially for screening tools—to increase their wide adoption.

## Figures and Tables

**Table 1:** Screening, diagnostic, and prognostic biomarkers, with the method used, sensitivity, specificity, and HR.

Intended Use	Biomarker	Test	Method	Sensitivity (95% CI) *	Specificity (95% CI) *	HR (95% CI)
Screening	sDNA	sDNA-FIT (Cologuard^®^) [20]	KRAS, NDRG4, BMP3, β-actin, and hemoglobin immunoassay	92.3% (83.0–97.5)	86.6% (85.9–87.2)	-
		Next-generation multitarget stool DNA (Cologuard Plus) [23]	LASS4, LRRC4, PPP2R5C, ZDHHC1, and hemoglobin immunoassay	94% (87.1–97.7)	90.6% (90.1–91.0)	-
	FIT [20]	-	Antibody-antigen reactions to the hemoglobin	73.8%; (61.5–84.0)	94.9% (94.4–95.3)	-
	mSEPT9	Epi procolon^®^ [26]	Septin 9 DNA	48.2% (32.4–63.6)	-	-
		-	mSEPT9 + CEA + CA 19-9 [28]	78.43% (upper limit 84.89%)	86.07% (lower limit 90.62%)	-
	Galectins family	-	Galectin-3 ligand [14]	70–80%	90% (AUC 0.87 to 0.90)	-
			Galectin-4 [31]	82.1%	61.2% (AUC 0.74)	-
			Galectin-4 + Tetraspanin 8 [31]	92.54%	67.16% (AUC 0.862)	-
Diagnostic	RAS, BRAF, and EGFR	OncoBEAM [40]	KRAS + NRAS genes	93% agreement	kappa index 0.844 (95% CI, 0.746–0.941)	-
		Idylla [41]	KRAS	93.3% agreement	-	-
			NRAS	94.4% agreement	-	-
	ctDNA	CancerSEEK [44]	multiplex-PCR DNA testing + 41 protein biomarkers	65% (60–70)	84–100% (accuracy of prediction)	-
	Multimodal blood-based test [45]	-	ctDNA-based blood + genomics + epigenomics + fragmentomics	93%	90%	-
	CTC	CellSearch [49]	≥2 tumoral cells	30%	-	-
	Multiplex CTC [50]	-	FCM + CellSearch + CK19 + MUC1 + CD44 + CD133 + ALD1	68.3%	95%	-
	GMSMs	-	8 GMSMs [52]	83.5%	84.8% (AUC 0.92)	-
		-	Fn-IgA + CEA + CA 19-9 [54]	40%	94.22% (AUC 0.74)	-
		-	Bf [53]	-	-	3.85 (2.62–5.64)
		-	Fn [53]	-	-	6.89 (1.70–27.9)
		-	Cs [53]	-	-	17.1 (1.82–160)
Prognostic	CEA [86]	-	Antigen assay of 2.5 µg/L	82%	80%	-
	CA 19-9 [87]	-	Antigen assay	81.8%	20%	-
	CA 125 [63]	-	Antigen assay	-	-	2.48 (1.68–3.65)
	CA 242 [63]	-	Antigen assay	-	-	3.23 (2.13–4.91)
	CSV-CTCs [66]	-	Cell surface vimentin-circulating tumor cells assay	-	-	3.78 (1.55–9.26)
	ctDNA [72]	-	TP53 + APC genes	-	-	10 (7.7–14)

* If not otherwise specified; Abbreviations: confidence interval (CI); hazard ratio (HR); multi-target stoold DNA (sDNA); fecal immunochemical testing (FIT); kirsten rat sarcoma virus (KRAS); N-myc downregulated gene 4 (NDRG4); bone morphogenetic protein 3 (BMP3); ceramide synthase 4 (LASS4); leucine-rich repeat-containing protein 4 (LRRC4); serine–threonine protein phosphatase 2A 56-kDa regulatory subunit gamma isoform (PPP2R5C); zinc finger DHHC-type containing 1 (ZDHHC1), methylated SEPTIN 9 (mSEPT9); carcinoembryonic antigen (CEA), carbohydrate antigen (CA); rat sarcoma virus (RAS); V-Raf Murine Sarcoma Viral Oncogene Homolog B (BRAF); epidermal growth factor receptor (EGFR); Kirsten rat sarcoma virus (KRAS); Neuroblastoma ras viral oncogene homolog (NRAS); polymerase chain reaction (PCR); circulating tumor DNA (ctDNA); circulating tumor cell (CTC); flowcytometry (FCM); Cytokeratin (CK); Mucin (MUC); cluster of differentiation (CD); aldolase (ALD); gut microbiome-associated serum metabolites (GMSM); Fusobacterium nucleatum (Fn); Bacteroides fragilis (Bf); Clostridium septicum (Cs); cell surface vimentin (CSV); tumor protein (TP); adenomatous polyposis coli (APC).
